# Enhanced recovery after vascular surgery: protocol for a systematic review

**DOI:** 10.1186/2046-4053-1-52

**Published:** 2012-11-02

**Authors:** Lesley Gotlib Conn, Ori D Rotstein, Elisa Greco, Andrea C Tricco, Laure Perrier, Charlene Soobiah, Tony Moloney

**Affiliations:** 1 Department of Surgery, St. Michael’s Hospital, 30 Bond Street, Toronto, ON, M5B 1W8, Canada; 2 Knowledge Translation Program, Li Ka Shing Knowledge Institute at St. Michael’s Hospital, 30 Bond Street, Toronto, ON, M5B 1W8, Canada; 3 Office of Continuing Education and Professional Development, Faculty of Medicine, University of Toronto, Toronto, Canada

**Keywords:** Vascular surgery, Enhanced recovery after surgery, ERAS, Fast-track, Systematic review

## Abstract

**Background:**

The enhanced recovery after surgery (ERAS) programme is a multimodal evidence-based approach to surgical care which begins in the preoperative setting and extends through to patient discharge in the postoperative period. The primary components of ERAS include the introduction of preoperative patient education; reduction in perioperative use of nasogastric tubes and drains; the use of multimodal analgesia; goal-directed fluid management; early removal of Foley catheter; early mobilization, and early oral nutrition. The ERAS approach has gradually evolved to become the standard of care in colorectal surgery and is presently being used in other specialty areas such as vascular surgery. Currently there is little evidence available for the implementation of ERAS in this field. We plan to conduct a systematic review of this literature with a view to incorporating ERAS principles into the management of major elective vascular surgery procedures.

**Methods:**

We will search EMBASE (OVID, 1947 to June 2012), Medline (OVID, 1948 to June 2012), and Cochrane Central *Register* of Controlled Trials (Wiley, Issue 1, 2012). Searches will be performed with no year or language restrictions. For inclusion, studies must look at adult patients over 18 years. Major elective vascular surgery includes carotid, bypass, aneurysm and amputation procedures. Studies must have evaluated usual care against an ERAS intervention in the preoperative, perioperative or postoperative period of care. Primary outcome measures are length of stay, decreased complication rate, and patient satisfaction or expectations. Only randomized controlled trials will be included.

**Discussion:**

Most ERAS approaches have been considered in the context of colorectal surgery. Given the increasing use of multiple yet different aspects of this pathway in vascular surgery, it is timely to systematically review the evidence for their independent or combined outcomes, with a view to implementing them in this clinical setting. Results from this review will have important implications for vascular surgeons, anaesthetists, nurses, and other health care professionals when making evidenced-based decisions about the use of ERAS in daily practice.

## Background

The enhanced recovery after surgery (ERAS) programme is a multimodal evidence-based approach to surgical care which begins in the preoperative setting and extends through to patient discharge in the postoperative period. This interprofessional, goal-directed pathway, involves surgeons, nurses, anaesthetists, and physiotherapists. It aims to accelerate the recovery of surgical patients while decreasing complications and reducing hospital length of stay. Since the early 1990s, the ERAS approach has gradually evolved to become the standard of care in colorectal surgery [1]. Advanced primarily by the Danish surgeon Dr. Henrik Kehlet [1-9], concepts in ERAS or fast-track surgery for colorectal patients have been studied and implemented internationally [10-15]. Evidence from randomized controlled trials suggests that ERAS protocols effectively and safely hasten the recovery of patients undergoing surgical colorectal procedures [14,16-18]. The primary components of ERAS include the introduction of preoperative patient education; reduction in perioperative use of nasogastric tubes and drains; the use of multimodal analgesia; goal-directed fluid management; early removal of Foley catheter; early mobilization, and early oral nutrition.

The use of ERAS protocols is reported in other surgical specialty areas, such as vascular [19,20], cardiac [21] and orthopaedic surgery [22-24]. In vascular surgery, ERAS implementation is reported for aortic [19,20,25-28] and carotid procedures [29]. Observational studies of elective vascular surgery patients (abdominal aortic aneurysm repair, aortobifemoral bypass grafting) using enhanced recovery pathways demonstrate reduced length of stay when compared with usual care [25,26,28]. A recent limited review of ERAS in open upper abdominal and thoracoabdominal surgery finds three studies evaluating ERAS in abdominal aortic surgery with positive outcomes for use of early oral nutrition and mobilization [30].

While there are a handful of general reviews on the topic of ERAS in vascular surgery [27,31,32] there is relatively little evidence for the use of an ERAS pathway in vascular surgery [33]. A systematic review of the literature is therefore timely with a view to incorporating ERAS principles into the management of major elective vascular surgery procedures. Our research question is: in adults over 18 years, what is the impact of enhanced recovery after surgery procedures in major elective vascular surgery in terms of reduced hospital length of stay, decreased complications, and improved patient expectations?

## Methods/Design

Eligibility: We will include studies of adult patients over the age of 18 who have undergone major elective vascular surgery and received an ERAS intervention. For this review major elective vascular surgery is defined as a carotid, aneurysm, bypass, or amputation procedure (see Appendix I). Studies must have evaluated the use of one or more ERAS intervention against usual care for patients (Table 1). Only randomized controlled trials will be included.

**Table 1:** ERAS interventions

**Preoperative**	**Perioperative**	**Post-operative**
Patient counseling/education	Abdominal drains (none)	Regular diet
Fasting (reduced)	Nasogastric tube (none)	Early feeding
e.g., Use of non-steroidal anti-inflammatory drugs (NSAIDs)	Fluid restriction	Pain management
Carbohydrate enriched drinks	Analgesia – NSAIDS, narcotics, use of epidural analgesia	Chewing gum/Ileus
Stress management	Fluid restriction	Early ambulation
Patient expectations (managing)		Exercise therapy
		Patient discharge planning
Accelerated rehabilitation	Urinary catheter (time of removal)	Removal of epidural catheter

### Search strategy

The databases searched will include: EMBASE (OVID, 1947 to June 2012), Medline (OVID, 1948 to June 2012), and Cochrane Central Register of Controlled Trials (Wiley, Issue 1, 2012). The database search will be supplemented by searching for grey literature (i.e. difficult to locate or unpublished material). Specifically, we will search online (e.g., Google), trial registers (e.g., Prospero), and conference abstracts. Searches will be performed with no year or language restrictions. Relevant non-English language articles will be translated. Literature search strategies will be developed by combining medical subject headings (MeSH terms) and appropriate wildcards. If necessary we will contact study authors to identify additional studies. The literature search will be conducted by an information specialist (LP) and peer-reviewed by another information specialist using Peer Review of Electronic Search Strategy (PRESS) [34]. In addition, we will hand search the references of selected studies. See Appendix II.

### Study selection

A pilot test of 50 randomly selected citations will be conducted by all authors to verify the inclusion/exclusion criteria. Subsequently, all studies (citations and full text) will be reviewed by two reviewers independently. Conflicts will be resolved by team discussion.

### Data collection

Data from included studies will be abstracted in duplicate by two reviewers using a data collection form. The data collection form will include:

a. patient characteristics (e.g., mean age, percent gender, number of patients)

b. study characteristics (e.g., study design, country of conduct, randomization procedure)

c. details of the intervention (e.g., which ERAS intervention was delivered – patient education, fluid restriction, early mobilization, etc.)

d. details of the comparator (e.g., placebo, usual care)

e. primary outcome results (e.g., length of stay, complications rate, patient expectations).

The data abstraction form will be pilot tested on a random sample of studies to ensure high inter-rater agreement between reviewers.

### Risk of bias assessment (quality)

Two reviewers will independently assess each selected study for the strengths of the research methods and results using the Cochrane Risk of Bias Tool [35]. This tool is comprised of the following six items: selection bias (sequence generation and allocation concealment), performance bias (blinding of participants and personnel), detection bias (blinding of outcome assessment), attrition bias (incomplete outcome data), selective outcome reporting, and other sources of bias. Disagreements will be discussed and resolved by consensus.

### Data synthesis

The results of the literature search will be presented in a flow diagram indicating the number of studies screened, the number of studies excluded, and reasons for exclusions. A synthesis of patient and study characteristics will be provided in separate tables including study citation and collected data items. A synthesis of risk bias results will be presented in a table format for each study as well as across studies. Primary outcomes will be synthesized in table format, including a summary of data for each intervention group, effect estimates, and confidence intervals. A separate subgroup analysis will be conducted for length of stay (LOS) based on the procedure and grouped by: endovascular, abdominal approach, extremity, and amputation. Complication-type will also be grouped by procedure (Table 2). To account for differences between interventions subgroups analyses by intervention will be conducted. If the interventions are too varied, data will not be pooled. If deemed appropriate, a random effects meta-analysis will be conducted. This will be conducted by type of surgery and individual ERAS intervention. Statistical heterogeneity will be examined using the I^2^ statistic [36].

**Table 2:** Complications

**Procedure**	**Immediately Post-operative**	**Within 30-days**
Carotid	TIA/CVA Bleeding Cranial Nerve injury –VII (Marginal Mandibular branch), X, XII Cardiovascular (myocardial infarction; Arrhythmias, Hypotension, Hypertension) Reperfusion Injury Infection (respiratory; urinary tract; surgical site)	Immediately Post-operative complications plus: Mortality Delayed Infection Pseudoaneurysm Cranial hemorrhage Recurrent Carotid Stenosis
Aneurysm (Open & Endovascular)	Cardiovascular (myocardial infarction; stroke; Arrhythmias) Renal failure Surgical site hematoma or bleeding Infection (respiratory; urinary tract; surgical site) Wound Dehiscence Pulmonary embolism/Deep Vein Thrombosis Endoleak (specific to endovascular repair)	Immediately post-operative complications plus: Open Mortality Nerve injury (Sexual dysfunction/ED) Incisional Hernia Small Bowel Obstruction Aorto-duodenal fistula Graft Infection Occlusion/Thrombosis Recurrent Aneurysm Endovascular Mortality Graft Infection Lymph Leak Endoleak +/- need for re-intervention Recurrent/Enlarging AAA Aorto-duodenal fistula
Bypasses	Cardiovascular (myocardial infarction; stroke; Arrhythmias) Infection (respiratory; urinary; surgical site) Leg edema Hemorrhage Reperfusion Injury Neuropraxia (nerve palsy) Pulmonary embolism/Deep vein Thrombosis Graft occlusion Graft infection	Immediately post-operative complications plus: Mortality Nerve injury (Sexual dysfunction/ED) Neurogenic pain Amputation
Amputation	Cardiovascular (myocardial infarction; heart failure; stroke; arrhythmia; renal failure) Poor/failed healing of stump Infection (respiratory; urinary; surgical site) Incisional stump pain Hematoma Falls	Immediately post-operative complications plus: Phantom limb pain Mortality Stump edema Dehiscence Tissue necrosis Osteomyelitis Pressure necrosis at site of bone transection

## Discussion

ERAS involves specified interventions at preoperative, perioperative, and postoperative points of care. Most ERAS approaches have been considered in the context of colorectal surgery. Given the increasing use of multiple yet different aspects of this pathway in vascular surgery, it is timely to systematically review the evidence for their independent or combined outcomes, with a view to implementing them in this clinical setting. We will disseminate the results of this review in an open access scientific journal and will present results at relevant scientific conferences. We expect our results will have important implications for vascular surgeons, anaesthetists, nurses, and other health care professionals when making evidenced-based decisions about the use of ERAS in their daily practice.

## Appendix

### Appendix I: Major elective vascular procedures

1. Carotid endarterectomy; carotid surgery.

2. Aneurysm: open and endovascular abdominal aortic aneurysm (AAA) surgery/repair (EVAR); infrarenal aneurysm surgery; thoracic aortic aneurysm surgery; thoraco-abdominal aortic aneurysm surgery; advanced EVAR; branched EVAR; fenestrated EVAR.

3. Bypass: supra-inguinal; infra-inguinal; femoral-femoral crossover; aorto-bi-iliac; aorto-uni-iliac (AUI); Aorto-Bifemoral bypass (ABF); Aorto-Femoral bypass; Ilio-Femoral bypass; Axillo-Femoral bypass; Axillo-BiProfunda bypass; Axillo-Bifemoral bypass; Femoro-Popliteal bypass; Femoro-Popliteal Above knee bypass; Femoro-Popliteal Below knee bypass; Femoro-Distal bypass; Femoro-Anterior tibial bypass; Femoro-Posterio tibial bypass; Femoro-Peroneal bypass; Popliteal-Pedal bypass; Common femoral repair; Femoral Endarterectomy; Remote Endarterectomy; Profundoplasty; Extended profundoplasty; Popliteal Artery Aneurysm; Common Femoral Aneurysm.

4. Amputation: Above knee amputation; Through knee amputation; Below knee amputation; Symes amputation; Transmetatarsal amputation.

### Appendix II: Literature search terms

Database: Ovid MEDLINE(R), Ovid MEDLINE(R) In-Process & Other Non-Indexed Citations, Ovid MEDLINE(R) Daily and Ovid OLDMEDLINE(R) <1946 to Present>

Search Strategy:

1. exp Vascular Surgical Procedures/[Vascular Surgery]

2. Aortic Aneurysm, Abdominal/su

3. Aortic Aneurysm, Thoracic/su

4. Endarterectomy, Carotid/

5. Endarterectomy/

6. (aneurysm adj repair$).tw.

7. (aneurysm adj surg$).tw.

8. (aort$ adj aneurysm$).mp.

9. (aort$ adj3 repair$).tw.

10. (aort$ adj3 bypass$).tw.

11. (aort$ adj3 by-pass$).tw.

12. (aort$ adj reconstruction$).tw.

13. (aort$ adj surg$).tw.

14. (arter$ adj aneurysm).tw.

15. (arter$ adj bypass$).tw.

16. (arter$ adj by-pass$).tw.

17. (arter$ adj repair$).tw.

18. (arter$ adj surg$).tw.

19. (axillo$ adj2 bypass$).tw.

20. (axillo$ adj2 by-pass$).tw.

21. ABF.tw.

22. AUI.tw.

23. AKA.tw.

24. (AAA adj repair$).tw.

25. (AAA adj surg$).tw.

26. (bypass$ adj surg$).tw.

27. (by-pass$ adj surg$).tw.

28. BKA.tw.

29. (carotid adj surg$).tw.

30. CEA.tw.

31. endarterectom$.tw.

32. (endovascular adj repair$).tw.

33. (endovascular adj surg$).tw.

34. (femor$ adj aneurysm).tw.

35. (femor$ adj repair$).tw.

36. (femor$ adj2 crossover$).tw.

37. (femor$ adj2 cross-over$).tw.

38. (femor$ adj3 bypass$).tw.

39. (femor$ adj3 by-pass$).tw.

40. (foot adj amputat$).tw.

41. "ilio femor$ bypass$".tw.

42. "ilio femor$ by-pass$".tw.

43. (iliofemoral adj bypass$).tw.

44. (iliofemoral adj by-pass$).tw.

45. (infrarenal adj aneurysm$).tw.

46. (infra-inguinal adj bypass$).tw.

47. (infra-inguinal adj by-pass$).tw.

48. (infrainguinal adj bypass$).tw.

49. (infrainguinal adj by-pass$).tw.

50. (knee adj1 amputat$).tw.

51. (leg adj revascular$).tw.

52. "popliteal pedal bypass$".tw.

53. "popliteal pedal by-pass$".tw.

54. profundoplast$.tw.

55. (supra-inguinal adj bypass$).tw.

56. (supra-inguinal adj by-pass$).tw.

57. (suprainguinal adj by-pass$).tw.

58. (suprainguinal adj bypass$).tw.

59. (symes adj amputat$).tw.

60. (tibia$ adj bypass$).tw.

61. (tibia$ adj by-pass$).tw.

62. (transmetatarsal adj amputat$).tw.

63. (TAA adj repair$).tw.

64. (TAA adj surg$).tw.

65. TEVAR.tw.

66. (vascular adj bypass).tw.

67. (vascular adj by-pass).tw.

68. (vascular adj surg$).tw.

69. (vascular adj repair$).tw.

70. (vascular adj reconstruction$).tw.

71. or/1-70

72. Chewing Gum/[Enhanced Recovery]

73. Early Ambulation/

74. Exercise Therapy/

75. Heating/

76. Intraoperative Care/mt

77. Preoperative Care/mt

78. Perioperative Care/mt

79. Postoperative Care/mt

80. Patient Education as Topic/

81. Surgical Procedures, Minimally Invasive/

82. exp Anesthesia/

83. an?esthesia.tw.

84. an?esthetic?.tw.

85. (accelerat$ adj2 mobil$).tw.

86. (accelerat$ adj2 ambulat$).tw.

87. (accelerat$ adj2 walk$).tw.

88. (accelerat$ adj2 feed$).tw.

89. (accelerat$ adj2 nutrition$).tw.

90. (accelerat$ adj2 eat$).tw.

91. (accelerat$ adj2 rehab$).tw.

92. (chew$ adj1 gum?).tw.

93. (client$ adj educat$).tw.

94. (client$ adj teach$).tw.

95. (client$ adj counsel$).tw.

96. (client$ adj expectation$).tw.

97. (crystalloid adj manage$).tw.

98. (crystalloid adj admin$).tw.

99. (earl$ adj2 mobil$).tw.

100. (earl$ adj2 ambulat$).tw.

101. (earl$ adj2 walk$).tw.

102. (earl$ adj2 feed$).tw.

103. (earl$ adj2 nutrition$).tw.

104. (earl$ adj2 eat$).tw.

105. (earl$ adj2 rehab$).tw.

106. (enhanced adj recover$).tw.

107. ERAS.tw.

108. (fast adj tract$).tw. (12)

109. (fast adj track$).tw. (1787)

110. (heat$ adj2 patient$).tw. (440)

111. intraoperative.mp. and (intravenous adj fluid?).tw. (207)

112. intraoperative.mp. and (IV adj fluid?).tw. (75)

113. intraoperative.mp. and Infusions, Intravenous/(856)

114. intraoperative.mp. and Intubation, Gastrointestinal/(123)

115. intraoperative.mp. and (NG adj tube?).tw. (5)

116. intraoperative.mp. and (nasogastric adj tube?).tw. (108)

117. intraoperative.mp. and (fluid? adj1 restrict$).tw. (59)

118. intraoperative.mp. and (abdominal adj drain$).tw. (35)

119. intraoperative.mp. and Anti-Inflammatory Agents, Non-Steroidal/241)

120. intraoperative.mp. and NSAID?.tw.

121. (intraoperative and analgesi$).mp.

122. (intraoperative and epidural?).mp.

123. (intraoperative and narcotic?).mp.

124. (intraoperative and (fluid adj therap$)).mp.

125. intraoperative.mp. and (fluid adj manag$).tw.

126. intraoperative.mp. and (electrolyte$ adj manag$).tw.

127. (intraoperative and (pain adj manage$)).mp.

128. (intraoperative and (vein adj thrombos?s)).mp.

129. ((intraoperative adj care) and enhanced).tw.

130. ((intraoperative adj care) and accelerat$).tw.

131. ((intraoperative adj care) and early).tw.

132. intra-operative.mp. and (intravenous adj fluid?).tw.

133. intra-operative.mp. and (IV adj fluid?).tw.

134. intra-operative.mp. and Infusions, Intravenous/

135. intra-operative.mp. and Intubation, Gastrointestinal/

136. intra-operative.mp. and (NG adj tube?).tw.

137. intra-operative.mp. and (nasogastric adj tube?).tw.

138. intra-operative.mp. and (fluid? adj1 restrict$).tw.

139. intra-operative.mp. and (abdominal adj drain$).tw.

140. intra-operative.mp. and Anti-Inflammatory Agents, Non-Steroidal/

141. intra-operative.mp. and NSAID?.tw.

142. (intra-operative and analgesi$).mp.

143. (intra-operative and epidural?).mp.

144. (intra-operative and narcotic?).mp.

145. (intra-operative and (fluid adj therap$)).mp.

146. intra-operative.mp. and (fluid adj manag$).tw.

147. intra-operative.mp. and (electrolyte$ adj manag$).tw.

148. (intra-operative and (pain adj manage$)).mp.

149. (intra-operative and (vein adj thrombos?s)).mp.

150. ((intra-operative adj care) and enhanced).tw.

151. ((intra-operative adj care) and accelerat$).tw.

152. ((intra-operative adj care) and early).tw.

153. (multimodal adj care).tw.

154. (multi-modal adj care).tw.

155. (patient$ adj educat$).tw.

156. (patient$ adj teach$).tw.

157. (patient$ adj counsel$).tw.

158. (patient$ adj expectation$).tw.

159. ((perioperative adj care) and enhanced).tw.

160. ((perioperative adj care) and accelerat$).tw.

161. ((perioperative adj care) and early).tw.

162. ((peri-operative adj care) and enhanced).tw.

163. ((peri-operative adj care) and accelerat$).tw.

164. ((peri-operative adj care) and early).tw.

165. postoperative.mp. and (regular adj diet?).tw.

166. postoperative.mp. and (normal adj diet?).tw.

167. postoperative.mp. and Enteral Nutrition/

168. (postoperative and catheter?).mp.

169. (postoperative and (fluid adj therap$)).mp.

170. postoperative.mp. and (fluid adj manage$).tw.

171. postoperative.mp. and (electrolyte$ adj manag$).tw.

172. (postoperative and analgesi$).mp.

173. (postoperative and opioid?).mp.

174. (postoperative and opiat$).mp.

175. (postoperative and epidural?).mp.

176. postoperative.mp. and Anti-Inflammatory Agents, Non-Steroidal/

177. postoperative.mp. and NSAID?.tw.

178. (postoperative and (pain adj manage$)).mp.

179. postoperative.mp. and (care adj map?).tw.

180. postoperative.mp. and (care adj plan$).tw.

181. postoperative.mp. and (treatment adj plan$).tw.

182. (postoperative and (clinical adj path$)).mp.

183. (postoperative and (care adj path$)).tw.

184. (postoperative and (critical adj path$)).mp.

185. (postoperative and (case adj management)).mp.

186. (postoperative and (patient adj discharge)).mp.

187. (postoperative and (discharge adj plan$)).mp.

188. (postoperative and (vein adj thrombos?s)).mp.

189. (postoperative and antiemetic?).mp.

190. (postoperative and anti-emetic?).mp.

191. (postoperative and ileus).mp.

192. ((postoperative adj care) and enhanced).tw.

193. ((postoperative adj care) and accelerat$).tw.

194. ((postoperative adj care) and early).tw.

195. post-operative.mp. and (regular adj diet?).tw.

196. post-operative.mp. and (normal adj diet?).tw.

197. post-operative.mp. and Enteral Nutrition/

198. (post-operative and catheter?).mp.

199. (post-operative and (fluid adj therap$)).mp.

200. post-operative.mp. and (fluid adj manage$).tw.

201. post-operative.mp. and (electrolyte$ adj manag$).tw.

202. (post-operative and analgesi$).mp.

203. (post-operative and opioid?).mp.

204. (post-operative and opiat$).mp.

205. (post-operative and epidural?).mp.

206. post-operative.mp. and Anti-Inflammatory Agents, Non-Steroidal/

207. post-operative.mp. and NSAID?.tw.

208. (post-operative and (pain adj manage$)).mp.

209. post-operative.mp. and (care adj map?).tw.

210. post-operative.mp. and (care adj plan$).tw.

211. post-operative.mp. and (treatment adj plan$).tw.

212. (post-operative and (clinical adj path$)).mp.

213. (post-operative and (care adj path$)).tw.

214. (post-operative and (critical adj path$)).mp.

215. (post-operative and (case adj management)).mp.

216. (post-operative and (patient adj discharge)).mp.

217. post-operative.mp. and (discharge adj plan$).tw.

218. (post-operative and (vein adj thrombos?s)).mp.

219. (post-operative and antiemetic?).mp.

220. (post-operative and anti-emetic?).mp.

221. (post-operative and ileus).mp.

222. ((post-operative adj care) and enhanced).tw.

223. ((post-operative adj care) and accelerat$).tw.

224. ((post-operative adj care) and early).tw.

225. (preoperative and fasting).mp.

226. preoperative.mp. and Anti-Inflammatory Agents, Non-Steroidal/

227. preoperative.mp. and NSAID?.tw.

228. preoperative.mp. and carbohydrate$.tw.

229. (preoperative and probiotic?).mp.

230. (preoperative and pro-biotic?).mp.

231. preoperative.mp. and hydrat$.tw.

232. preoperative.mp. and dehydrat$.tw.

233. preoperative.mp. and de-hydrat$.tw.

234. preoperative.mp. and stress.tw.

235. ((preoperative adj care) and enhanced).tw.

236. ((preoperative adj care) and accelerat$).tw.

237. ((preoperative adj care) and early).tw.

238. (pre-operative and fasting).mp.

239. pre-operative.mp. and Anti-Inflammatory Agents, Non-Steroidal/

240. pre-operative.mp. and NSAID?.tw.

241. pre-operative.mp. and carbohydrate$.tw.

242. (pre-operative and probiotic?).mp.

243. (pre-operative and pro-biotic?).mp.

244. pre-operative.mp. and hydrat$.tw.

245. pre-operative.mp. and dehydrat$.tw.

246. pre-operative.mp. and de-hydrat$.tw.

247. pre-operative.mp. and stress.tw.

248. ((pre-operative adj care) and enhanced).tw.

249. ((pre-operative adj care) and accelerat$).tw.

250. ((pre-operative adj care) and early).tw.

251. (rapid$ adj2 recover$).tw.

252. (rapid adj2 mobil$).tw.

253. (rapid adj2 ambulat$).tw.

254. (rapid adj2 walk$).tw.

255. (rapid adj2 feed$).tw.

256. (rapid adj2 nutrition$).tw.

257. (rapid adj2 eat$).tw.

258. (rapid adj2 rehab$).tw.

259. (warm$ adj patient$).tw.

260. or/72-259

261. 71 and 260

262. exp Animals/ not (Humans/ and exp Animals/)

263. 261 not 262 [ Removing Animal Studies ]

264. randomized controlled trial.pt. [ RCT filter - validated - optimized ]

265. randomized.mp.

266. placebo.mp.

267. or/264-266

268. 263 and 267

## Abbreviations

ERAS: Enhanced recovery after surgery; AAA: Abdominal aortic aneurysm; EVAR: Endovascular aneurysm repair; AUI: Aorto-uni-iliac; ABF: Aorto-Bifemoral bypass; NSAIDs: Non-Steroidal Anti-Inflammatory drugs; MeSH: Medical subject headings.

## Competing interests

There are no competing interests.

## Authors’ contributions

LGC collaborated in the design of the study and drafted the protocol. OR conceived of the study, collaborated in the design of the study, and edited the protocol. EG collaborated in the design of the study and edited the protocol. ACT collaborated in the design of the study and edited the protocol. LP developed the search strategy and edited the protocol. CS collaborated in the design of the study and edited the protocol. TM collaborated in the design of the study and edited the protocol. All authors read and approved the final manuscript.

## Funding

This systematic review is funded by the Department of Surgery, St. Michael’s Hospital. ACT is funded by a Canadian Institutes for Health Research/Drug Safety and Effectiveness Network New Investigator Award in Knowledge Synthesis.
